# Origin of perpendicular magnetic anisotropy in amorphous thin films

**DOI:** 10.1038/s41598-020-78950-7

**Published:** 2021-02-12

**Authors:** Daniel Lordan, Guannan Wei, Paul McCloskey, Cian O’Mathuna, Ansar Masood

**Affiliations:** 1 Micro & Nano Systems Centre, Tyndall National Institute, University College Cork, Lee Maltings, Dyke Parade, Cork, T12 R5CP Ireland; 2 Department of Electrical and Electronic Engineering, University College Cork, Cork, Ireland

**Keywords:** Materials for devices, Magnetic properties and materials

## Abstract

The emergence of perpendicular magnetic anisotropy (PMA) in amorphous thin films, which eventually transforms the magnetic spins form an in-plane to the out-of-plane configuration, also known as a spin-reorientation transition (SRT), is a fundamental roadblock to attain the high flux concentration advantage of these functional materials for broadband applications. The present work is focused on unfolding the origin of PMA in amorphous thin films deposited by magnetron sputtering. The amorphous films were deposited under a broad range of sputtering pressure (1.6–6.2 mTorr), and its effect on the thin film growth mechanisms was correlated to the static global magnetic behaviours, magnetic domain structure, and dynamic magnetic performance. The films deposited under low-pressure revealed a dominant in-plane uniaxial anisotropy along with an emerging, however feeble, perpendicular component, which eventually evolved as a dominant PMA when deposited under high-pressure sputtering. This change in the nature of anisotropy redefined the orientation of spins from in-plane to out-of-plane. The SRT in amorphous films was attributed to the dramatic change in the growth mechanism of disorder atomic structure from a homogeneously dispersed to a porous columnar microstructure. We suggest the origin of PMA is associated with the columnar growth of the amorphous films, which can be eluded by a careful selection of a deposition pressure regime to avoid its detrimental effect on the soft magnetic performance. To the author’s best knowledge, no such report links the sputtering pressure as a governing mechanism of perpendicular magnetisation in technologically important amorphous thin films.

## Introduction

The soft magnetic properties of thin films as a core material are critical for the realisation of future miniaturised electromagnetic devices, i.e. micro-transformers and micro-inductors, which essentially requires operation at high frequencies^1–5^. To achieve this level of miniaturisation, the pre-requisite of soft magnetic materials are high saturation magnetisation (*M*_*s*_), high permeability (*µ*), high electrical resistivity (*ρ*), negligible material loss at high frequencies, and most importantly, ease of manufacturability^6,7^ to integrate onto a silicon for a complete power supply on chip (PwrSoC) concept^8^. The state-of-the-art soft magnetic materials are amorphous metals that retain exceptional properties such as high *M*_*s*_ (10–18 kG), high *µ* (10^5^), ultra-low coercivity (*H*_*c*_ < 1 Oe) and high *ρ* (100–150 µΩ cm)^9,10^. These phenomenal attributes are due to the unique disorder atomic structure, similar to liquid melts, that retain a high electrical resistivity, zero magnetocrystalline anisotropy, near-zero magnetostriction, and most importantly, induced in-plane uniaxial anisotropy^11^, which makes the amorphous materials functional for a broad range of applications. Soft magnetic amorphous metals are well investigated in bulk form; however, the thin-film study of these novel alloys, while retaining their excellent properties for integrated magnetics, is rather a recent area of interest.

The fundamental roadblock of utilising soft magnetic amorphous films in broadband electromagnetic devices is perpendicular magnetic anisotropy (PMA)^12^. The PMA in thin films confines the magnetisation in an out-of-plane configuration, which eventually results in deteriorated soft magnetic properties, such as high *H*_*c*_, low relative permeability (*µ*_*r*_), and high material loss due to multimode resonance in the permeability spectrum^13^. Several mechanisms have been proposed as an origin of the PMA in amorphous films, such as high degree of atomic randomness, residual stress, nature of stress (compressive/tensile) and large magnetostriction of the alloys^14,15^. The PMA is known to strictly depend on film thickness, alloy composition, nature of fabrication process, substrate temperature under deposition and post-annealing conditions of films^14,16–19^. Furthermore, the same alloy seems to show different spin-orientation when deposited in different sputter chambers, suggesting its origin is based on deposition parameters and geometry of the sputter chamber. The dynamic nature of PMA, owning to aforementioned reasons, governs the spins from in-plane to out-of-plane direction, also well-known as a spin-reorientation transition (SRT), and has been witnessed in many amorphous thin film systems^20,21^. If the origin of SRT, why spins transforms from in-plane to out-of-plane configuration for the alloy when deposited under different sputter tools, is well-understood the amorphous films can be widely adopted in microelectronic industry for a broad range of soft magnetic applications, including PwrSoC^22^.

Amorphous thin films are usually deposited by physical vapour deposition (PVD) technique, such as magnetron sputtering with argon as a sputter gas^23^. The power applied to the target determines the deposition rate of the material, an important parameter to control the thin film growth mechanism. Another critical deposition parameter of sputtering to consider is the argon sputtering pressure. The argon pressure affects the energy and angle at which sputtered atoms arrive at the substrate and subsequently condense to form the film. At high pressures, the sputtered atoms suffer many collisions with the working gas and arrive at the substrate with reduced momentum, kinetic energy and larger incident angles^24^. The inverse is true for low pressures. In these two different pressure regimes, one would expect the microstructure of the films to differ. One has to consider how the amorphous material itself is deposited on the substrate, how it influences the nature of the underlying magnetism, and consequently, its effects on the ultra-soft magnetic properties for broadband drive applications. Most recently, a magnetic transition from superparamagnetism to spin glass behaviour of Fe–Zr thin films was correlated to a very slight change in microstructure of the amorphous films when deposited under different pressure^25^. To the author’s best knowledge, no such report links the sputtering pressure as a mechanism of SRT in amorphous thin films.

The present work is focused on the origin of mechanisms governing the PMA in amorphous thin films deposited by magnetron sputtering. It demonstrates how sputtering deposition pressure can influence the thin film growth mechanism of amorphous films from a homogeneously dispersed disorder atomic structure to a porous columnar microstructure, which, consequently, transform the spins from in-plane to out-of-plane configuration due to the associated PMA.

## Results and discussion

### Structural properties of as-deposited films

**Figure 1:**
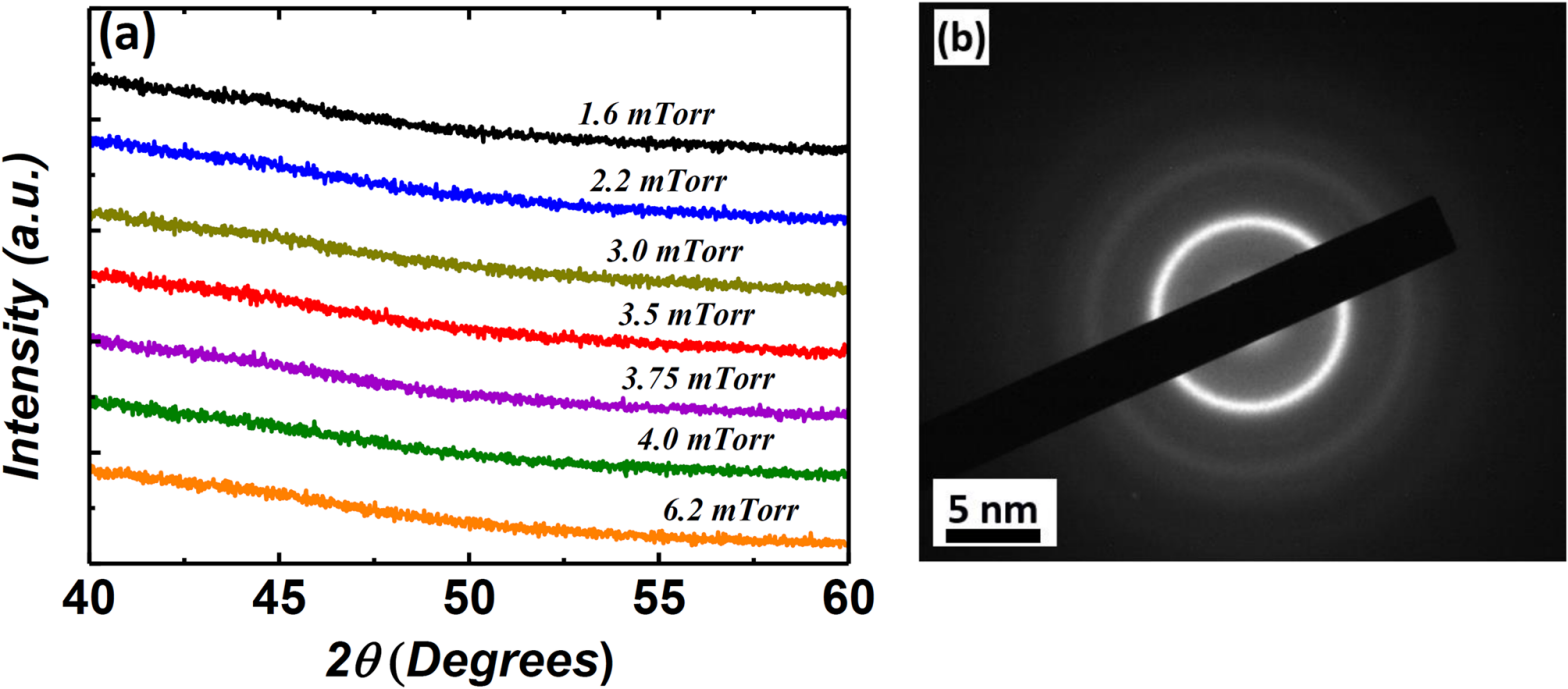
(**a**) The XRD spectrum of Co–Zr–Ta–B films deposited under different argon sputtering pressures (1.6–6.2 mTorr). (**b**) SAED pattern of the film deposited under 6.2 mTorr argon pressure.

The atomic structure of each film was analysed using x-ray diffraction (XRD) in the range of 2θ = 40°–60°. No sharp Bragg’s peak was observed for the films deposited under different argon pressures of 1.6–6.2 mTorr, as presented in Fig. 1. The absence of Bragg’s peak in the XRD spectrum shows that the atomic structure of films remained amorphous under various deposition pressures, which confirms there is no pressure-dependent change on the atomic structure of the films. The sputtering of films under high pressure can change the atomic structure, and hence, affects the optimum performance of thin-film materials. For example, reactively sputtered TiN films showed a transition from crystalline to a quasi-amorphous phase when the sputtering pressure was increased from 2 to 12 mTorr^26^. Furthermore, a selected area electron diffraction (SAED) pattern of the 6.2 mTorr film, Fig. 1b, showed a diffuse ring pattern, which further confirms the localised amorphous structure of the films deposited under highest deposition pressure. The amorphous structure of Co–Zr–Ta–B films is vital to retain high electrical resistivity, due to random electron scattering, and ultra-soft magnetic properties, due to absence of magnetocrystalline anisotropy, for high-frequency applications^13,27^.

The resistivity (*ρ*) of each film was measured using a 4-point probe in the centre of each wafer. Note that the resistivity of the titanium layer was subtracted for each film. All films show a high resistivity, which is critical to reducing eddy current losses^6^. Resistivity values were measured in the range of ~ 117–131 µΩ cm for the present series of films. A resistivity value of ~ 115 µΩ cm has been reported elsewhere for Co–Zr–Ta–B^28^. A small variation in the resistivity values might be due to the various assumptions considered during measurements. One such assumption is that the film thickness is homogenous across the whole sample. In addition, the resistivity is influenced by other microscopic features such as voids and rough surfaces, which can be introduced during film deposition^29^. A marginal variation in the resistivity of the films deposited under a broad pressure range further suggest the amorphous nature of the atomic structure at the local atomic scale, hence it is in good agreement with the XRD and SAED structural observations (Fig. 1).

**Figure 2:**
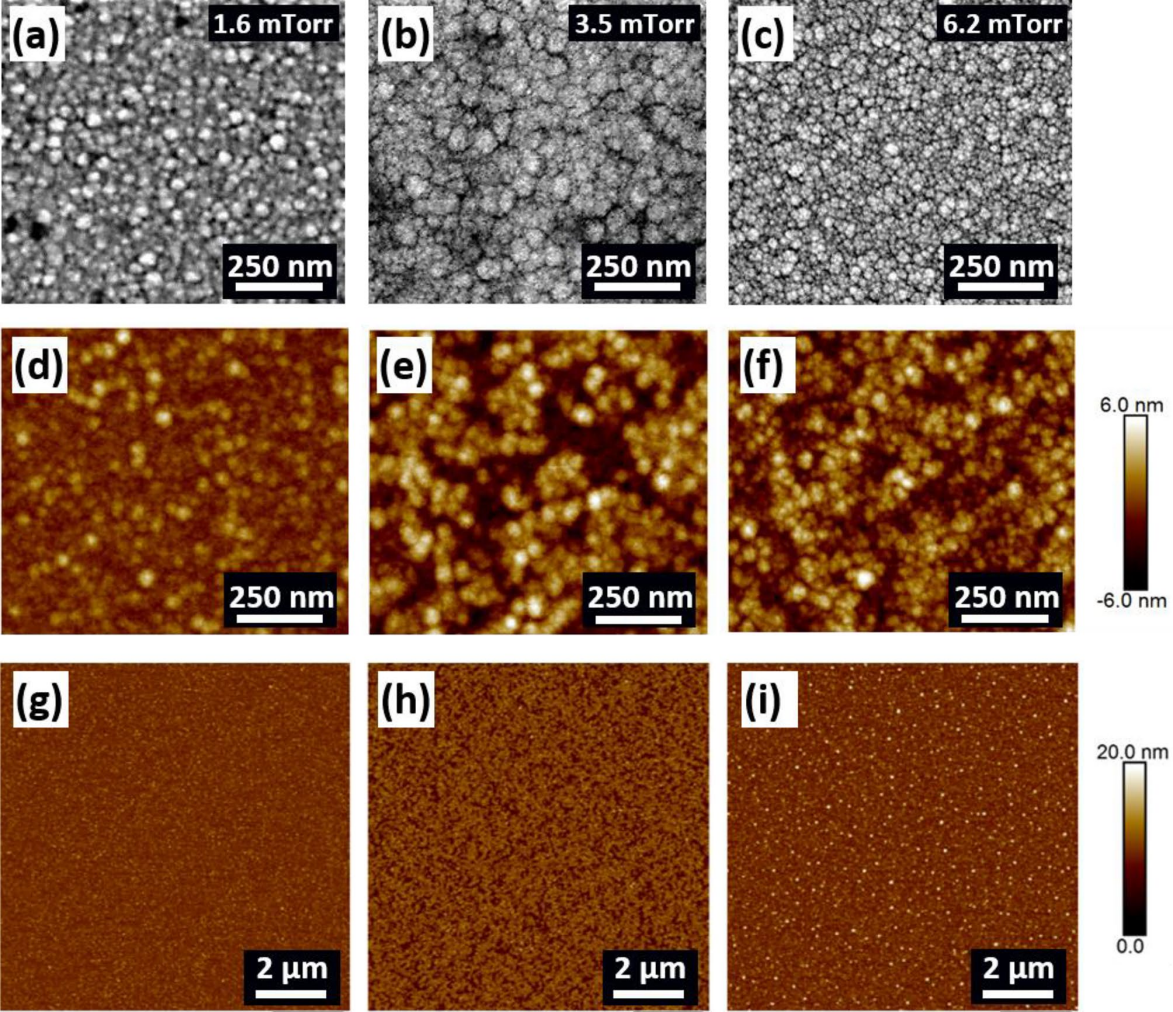
SEM images of the thin films deposited at (**a**) 1.6 mTorr, (**b**) 3.5 mTorr and (**c**) 6.2 mTorr along with corresponding AFM images in (**d**), (**e**), (**f**), (**g**), (**h**) and (**i**).

Scanning electron microscopy (SEM) images show the surface morphology of the 1.6 mTorr, 3.5 mTorr, and 6.2 mTorr pressure films, as presented in Fig. 2. The number of grains and grain size for each film was calculated using ImageJ analysis software^30^. The lower pressure film of 1.6 mTorr, see Fig. 2a, displays a dense microstructure of ~ 784 grains with an average grain size of ~ 40 nm (grain size herein was measured as the width of the grains). The 3.5 mTorr pressure film has ~ 208 grains with an average size of ~ 78 nm. Conversely, the higher-pressure film of 6.2 mTorr displays a much denser microstructure when compared to the other two films, as presented in see Fig. 2c. A total of ~ 1122 grains were counted for this film with an average grain size of ~ 34 nm. Further, atomic force microscopy (AFM) was performed over a similar-sized area, as presented in Fig. 2d–f, which revealed similar characteristics of the films. The grain size of the 1.6, 3.5 and 6.2 mTorr films (averaged over 10 grains) was found to be ~ 36 nm, 64 nm and 35 nm, respectively, from the AFM images. Later, a larger scan area (10 µm × 10 µm) was imaged to investigate surface topography. Root mean square roughness of 1 nm, 1.6 nm, and 1.2 nm were measured over the whole scan area for the 1.6 mTorr, 3.5 mTorr, and 6.2 mTorr pressure films, respectively. The small roughness values suggest quite a smooth surface of the films, as can be seen in Fig. 2g,h,i.

**Figure 3:**
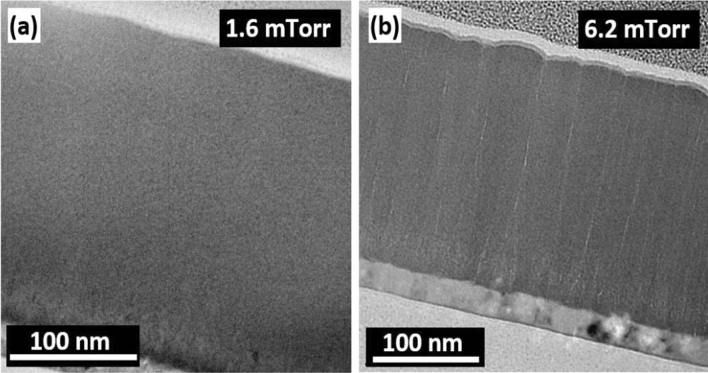
TEM image of the cross-section of 250 nm film deposited under different sputtering pressures, (**a**) 1.6 mTorr, (**b**) 6.2 mTorr.

Further, the thin film growth process was investigated by transmission electron microscopy (TEM) imaging of the cross-section of the films thinned by focused ion beam (FIB). Figure 3 presents the TEM micrographs of the films deposited at the lowest (i.e., 1.6 mTorr) and highest (i.e., 6.2 mTorr) deposition pressures. Ultra-smooth growth of films was observed in 1.6 mTorr pressure film, see Fig. 3a, thanks to the homogeneous disordered atomic structure that makes the amorphous films viable for a broad range of MEMS applications. Contrarily, the microstructure of film was remarkably distinct under high-pressure deposition, thus proposing a fundamental divergence in the mechanism of the thin-film growth process. Cross-sections of the film deposited under 6.2 mTorr is illustrated in Fig. 3b, suggesting that high-pressure deposition leads to a porous columnar growth structure. The size of the voided columnar regions measured using TEM is ~ 36 nm, which is on the same order of magnitude for the grain size estimated from the SEM (~ 34 nm) and the AFM (~ 35 nm), see Fig. 2c,f. The pressure-dependent change in the microstructure of amorphous films could cause a different nature of anisotropy energies to emerge, i.e., magnetostatic or magnetoelastic, that might be competitive enough to influence the global magnetic behaviours of the films, and, eventually, soft magnetic performance of the films.

The microstructure of sputtered films results from the energy of the sputtered atoms and the angles at which they arrive at the substrate^25,31^. At low pressures, the sputtered atoms have a long mean free path and overcome a shadowing effect during the initial nucleation of the film due to being highly energetic. This shadowing effect is an interaction between the initial roughness of the substrate and the angle at which sputtered atoms arrive at the substrate. Thus, in this low-pressure regime, the sputtered atoms from the Co–Zr–Ta–B arrive at the substrate with high energy, strike the material out, and consequently, no inter-columnar voids are produced. Diversely, for the higher argon pressure of the 6.2 mTorr, the mean free path of sputtered Co–Zr–Ta–B atoms is reduced due to scattering with the working argon gas and creates a bigger angle of incidence when arriving at the substrate. In this high-pressure regime, the sputtered atoms are less energetic when arriving at the substrate, and the material is unable to overcome the shadowing effect during the initial nucleation of the film. The material preferentially grows on higher surface features and prevents deposition on shadowed regions. This shadowing effect produces inter-columnar voids on the substrate, as the atoms are unable to be incorporated at grain boundaries^32^. This is in agreement with Thornton’s growth model of thin films, which suggests how high working gas pressure results in a porous columnar type growth structure due to this pinning effect during the initial nucleation of the film^33^. Kim et al.^25^, demonstrated that high pressure during sputtering lead to a porous columnar microstructure for amorphous Fe–Zr films, which eventually, transforms the nature of the underlying magnetism. The solidus melting temperature of the Co–Zr–Ta–B alloy was found as ~ 1021 °C by calculating the phase diagram using Pandat software (version 2019, https://computherm.com/)^34^. The temperature of the substrate during low pressure (1.6 mTorr) growth was measured as ~ 110 °C by using temperature sensitive labels. Note that this value is an upper bound. As the pressure increases, the atoms arrive at the substrate with less energy, decreasing the substrate temperature during deposition. This substrate temperature value results in a maximum *T*_*s*_/*T*_*m*_ ratio of ~ 0.1, and thus the high-pressure film is consistent with a Zone 1 type growth formation from the Thornton model. Zone 1 type growth is associated with the formation of a columnar microstructure with voided boundary regions and occurs when the ratio of *T*_*s*_/*T*_*m*_ is low in conjunction with an elevated working gas pressure^35^. The absence of porous columns in the low-pressure films is caused by the coating atoms having sufficient energy to fill the voided regions due to lack of collisions with the working gas, causing the coating atoms to arrive at near normal incidence. The pinning of sputtered atoms during the initial nucleation of the film may explain why the high pressure regime film (i.e. 6.2 mTorr) has a larger number of grains as seen in the SEM and AFM images in Fig. 2c,f.

### DC magnetic properties

**Figure 4:**
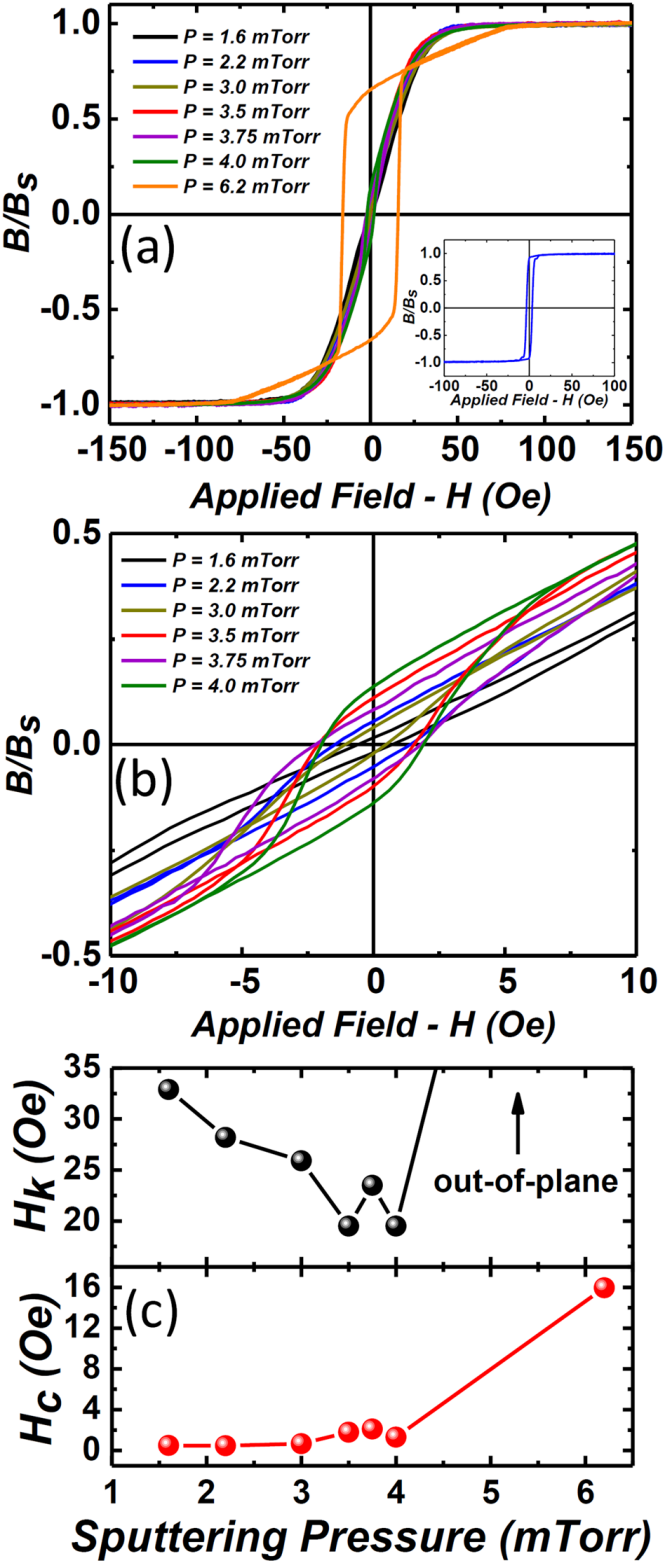
(**a**) BH loops of Co–Zr–Ta–B thin films deposited under different argon sputtering pressure. Inset shows the easy axis hysteresis loop of the 1.6 mTorr film, (**b**) Zoomed-in area of the BH loops of films deposited at 1.6–4 mTorr pressure showing the evolution of perpendicular magnetic component, (**c**) Anisotropic filed (*H*_*k*_) and coercivity (*H*_*c*_) versus argon sputtering pressure for all films.

Figure 4a presents the BH loops of the films measured along the hard anisotropy axis, while the inset shows a typical easy anisotropy axis BH loop of the film deposited under 1.6 mTorr pressure. A well-defined easy and hard anisotropy axis of BH loops show that a significant uniaxial anisotropy was induced during the deposition process of films deposited in low-pressure regime (1.6–4 mTorr). Contrarily, the nature of magnetic anisotropy was surprisingly different in the high-pressure films (i.e., 6.2 mTorr). Interestingly, the zoomed-in region of hard anisotropy loops of low-pressure films (1.6–4 mTorr), presented Fig. 4b, show two distinct slopes before saturation. Nevertheless, the strength of the slope, i.e. close to zero-field, is feeble as compared to the 6.2 mTorr film. More importantly, the slope of the loops continuously emerged as a function of deposition pressure, thus suggesting a pressure-dependent evolution in the nature of magnetic anisotropy. Furthermore, the effect of the evolution of magnetic anisotropy is evident in the resultant anisotropic field (*H*_*k*_) of the films measured using the BH loops; see Fig. 4c. The highest uniaxial *H*_*k*_ ~ 32.92 Oe was measured for the 1.6 mTorr film. The *H*_*k*_ values decreased as a function of increasing pressure, due to the competing uniaxial and perpendicular components, and approached a minimum *H*_*k*_ ~ 19.51 Oe in the pressure range of 3.5–4 mTorr. The dramatic increase in *H*_*k*_ ~ 75 Oe of 6.2 mTorr film was due to the isotropic BH loop with no discernible easy or hard magnetic axis ascribed to the dominant PMA component. Another evolution in the coercivity (*H*_*c*_) of films was observed, which steadily increased with sputtering pressure with a minimum *H*_*c*_ ~ 0.49 Oe at 1.6 mTorr to an undesirable large *H*_*c*_ ~ 15.95 Oe for 6.2 mTorr pressure (Fig. 4c).

A double-sloped hysteresis, known as a transcritical loop, is indicative of perpendicular magnetisation, where the slope emerging before the saturation shows the PMA component^36^. Films in the pressure range of 1.6–4 mTorr display both uniaxial in-plane anisotropy and a PMA, as evident from the double-sloped loops presented in Fig. 4b. It further illustrates that the PMA component is deposition pressure-dependent, and once the pressure was increased from 4 to 6.2 mTorr, the PMA component becomes dominant. The resultant hysteresis of the 6.2 mTorr films remains isotropic and displays a transcritical shape, which is attributed to the dominant out-of-plane magnetisation^37^. This phenomenon is known as SRT, where magnetisation transforms from in-plane to out-of-plane configuration and has been recently reported for many amorphous thin films^14,20^.

**Figure 5:**
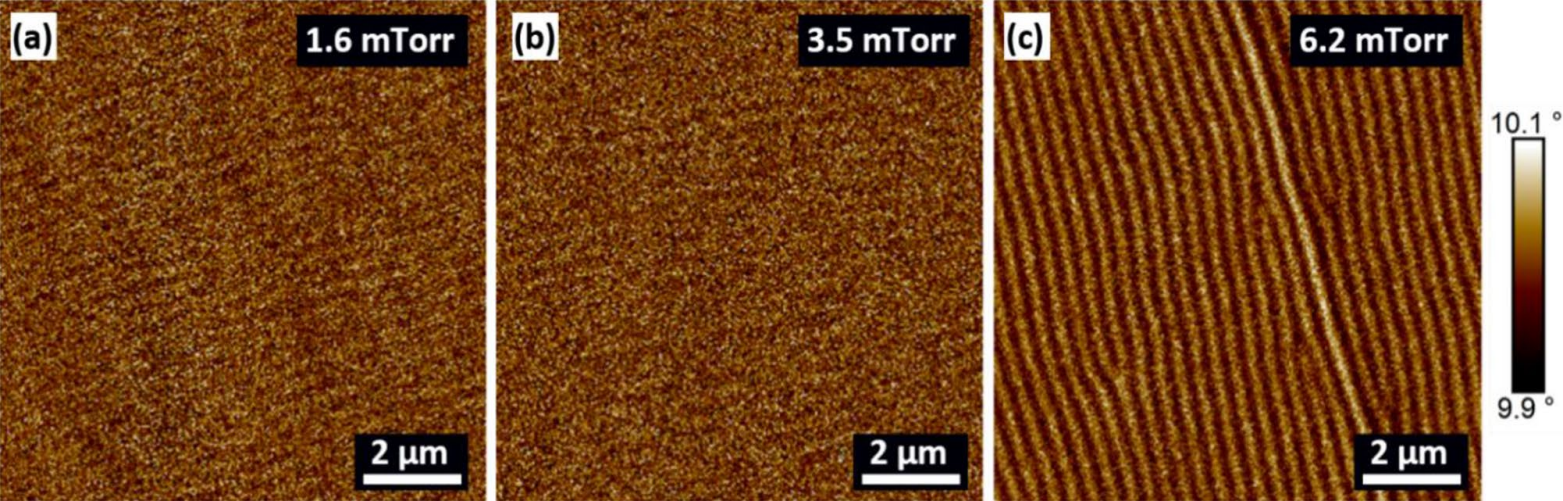
MFM images performed at the remnant state of magnetisation of the films deposited under different sputter pressure, (**a**) 1.6 mTorr, (**b**) 3.5 mTorr, (**c**) 6.2 mTorr.

Magnetic force microscopy (MFM) images were obtained at the remnant state of magnetisation for the following films; 1.6, 3.5 and 6.2 mTorr, as presented in Fig. 5. The 1.6 mTorr and 3.5 mTorr pressure films display some faint striped domain patterns, as seen in Fig. 5a,b. This is in good agreement with the BH loops that manifested a weak PMA component in the films deposited under low-pressure. However, once the pressure increased beyond the critical point to 6.2 mTorr, a distinct striped domain pattern emerged, as seen in Fig. 5c. A well-defined striped magnetic domain suggests that the easy axis magnetisation of the film resides perpendicular to the plane. The difference in contrast of striped domains is related to the orientation of the magnetic spins, which are pointing either upward or downward in relation to the film plane^36^. The change in sign of the magnetisation component between domains causes a reduction in the effective anisotropy energy and hence, explains why a higher external field (i.e., > 75 Oe) is required to saturate the 6.2 mTorr film^38,39^.

### AC magnetic properties

**Figure 6:**
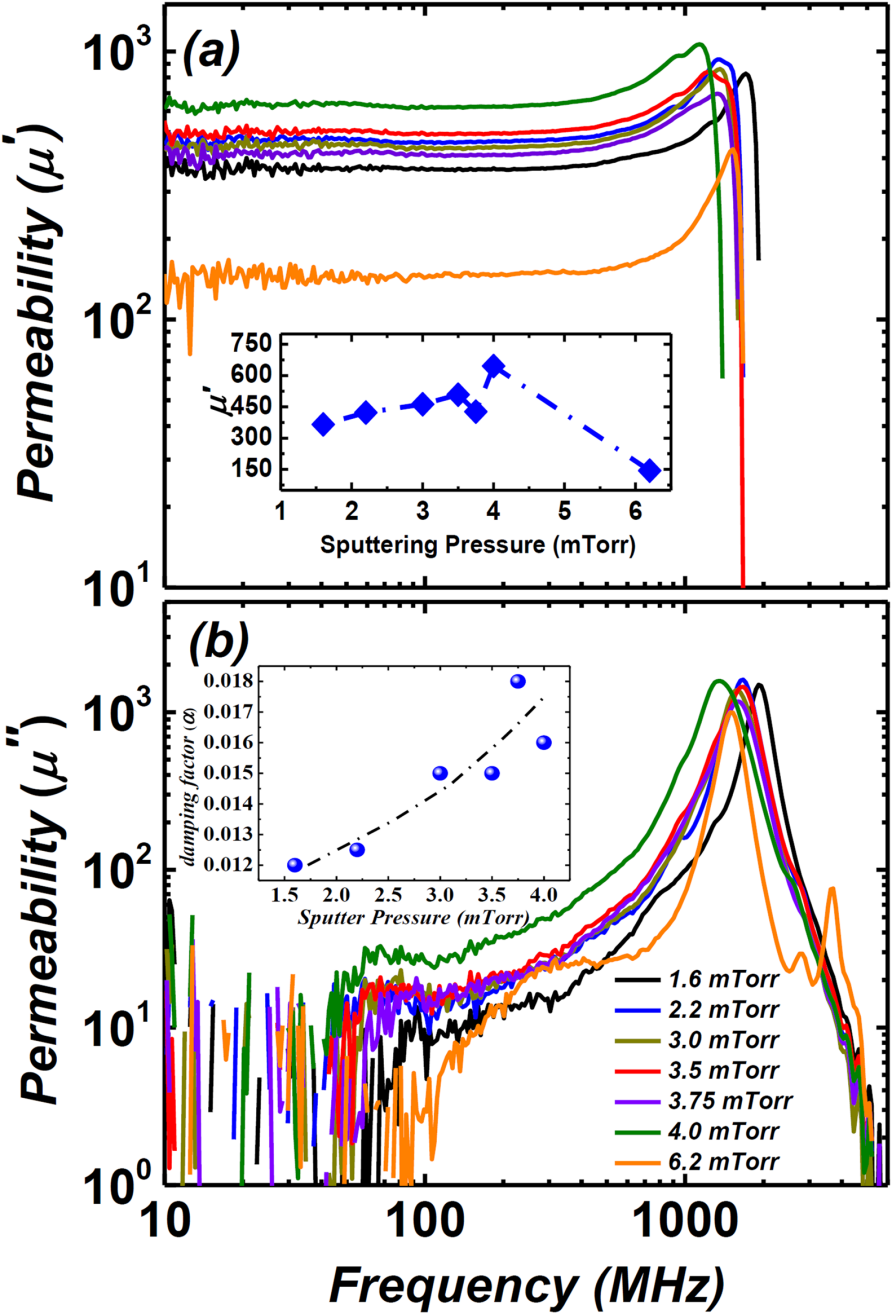
(**a**) High-frequency real permeability (*μʹ*) spectrum of for films deposited under different sputtering pressure (1.6–6.2 mTorr). The inset shows the values of *μʹ* at f = 40 MHz versus argon sputtering pressure. (**b**) High-frequency imaginary permeability (*μʹʹ*) for all films. Note the emergence of multiple peaks after the main $f$_FMR_ for the films deposited under the highest sputtering pressure (i.e., 6.2 mTorr) in the present series of films. The inset shows the Gilbert damping factor, α, of films deposited under different argon sputter pressures.

The permeability of the films was measured in the frequency range of 1 MHz—9 GHz, as presented in Fig. 6. The films deposited under low-pressure (1.6–4 mTorr) show high permeability and ferromagnetic resonance frequency (*f*_*FMR*_ > 1 GHz). This could be ascribed to the dominant uniaxial anisotropy induced during deposition pressure of the films, as seen from BH loops (Fig. 4). Inset Fig. 6a shows the real permeability values (*µ*ʹ, quoted at *f* = 40 MHz) as a function of Ar sputter pressure. The 1.6 mTorr film displayed the lowest *µ*^*ʹ*^ ~ 366 for low-pressure regime films and is related to the large *H*_*k*_ ~ 32.92 Oe. The 3.5 mTorr and 4 mTorr films having the lowest *H*_*k*_ ~ 19.5 Oe display the highest *µ*ʹ ~ 509 and ~ 646, respectively. The imaginary component of permeability (*µʺ*) of each film is negligibly small up to 100 MHz, as presented in Fig. 6b. The smaller *µʺ* is due to the thickness of the films, which is below the skin depth of the Co–Zr–Ta–B alloy^40^. Interestingly, a small kink at the shoulder of the main *f*_*FMR*_ was observed in all films, except for the 6.2 mTorr sample. The emergence of multiple peaks before the main FMR could be attributed to the heterogeneous amorphous structure of the films, as reported by the same authors elsewhere^13^. More interestingly, the 6.2 mTorr film displayed low *µ*ʹ ~ 144 and multiple resonance peaks after the main $f$_FMR_ both in the real and imaginary permeability spectra. The low *µ*ʹ and multiple peaks may have emerged due to the strong PMA component, as confirmed from BH loops and domain imaging.

The Landau–Lifschitz–Gilbert (LLG) equation (see Eq. 1) was used to calculate the Gilbert damping constant, *α,* for the films with dominant in-plane uniaxial anisotropy (i.e., films deposited under low deposition pressure)^41^

1 $$\mu =\left\{1+\frac{{\gamma }^{2}{M}_{s}\cdot \left({H}_{k}-{M}_{s}+\frac{i\alpha \omega }{\gamma }\right)}{{\gamma }^{2}{H}_{k}\cdot \left({H}_{k}+{M}_{s}\right)-{\omega }^{2}+i\omega \alpha \gamma \cdot \left(2{H}_{k}+4\pi {M}_{s}\right)}\right\}\cdot \left(\frac{tanh\left[\frac{\left(1+i\right)t}{2\delta }\right]}{\frac{\left(1+i\right)t}{2\delta }}\right)$$

where *M*_*S*_ is the saturation magnetisation, *H*_*k*_ is the anisotropy field, *γ* is the gyromagnetic ratio, *t* is the thickness of the film and *δ* is the skin depth as $\sqrt{2\uprho /\left(\upomega \cdot {\upmu }_{0}\cdot {\upmu }_{\mathrm{static}}\right)}.$ The damping factor, *α*, describes the ease of the spins to undergo magnetisation reversal at high frequencies^42^. One would expect the loss of the material to be substantial at high frequencies if the value of *α* is significantly large. The *α* increased gradually as a function of deposition pressure, as shown in Fig. 6b inset hence suggesting how emerging PMA, however feeble, is correlated to the damping loss of the films that could rise at high frequencies to undermine the performance of the material.

**Figure 7:**
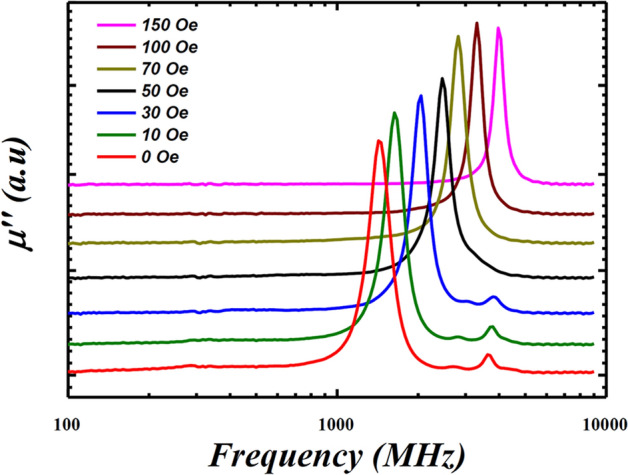
High-frequency imaginary permeability (*µʹʹ*) of the 6.2 mTorr films under a bias field from 0 to 150 Oe along easy anisotropy axis. The multiple resonance peaks after main FMR peak disappears when the bias field ≥ 70 Oe.

The high-frequency permeability spectrum strongly depends on the magnetic domain structures of the material. Multiple $f$_FMR_ peaks normally can originate from stripe magnetic domains due to perpendicular magnetisation^43^. The effect of magnetic domain structure on the permeability spectrum of the films can be negated, if any, to a single domain by saturating the films using a bias magnetic field. The in-plane biased magnetic field from 0 to 150 Oe was applied along the easy-axis of 6.2 mTorr film and response of multiple resonance peaks was investigated, as presented in Fig. 7. The multiple peaks only disappeared when the bias-field increased to 70 Oe, that is similar to the measured *H*_*k*_ ~ 75 Oe required to saturate the 6.2 mTorr film to a single magnetic domain (see Fig. 4a). This concludes that the multiple peaks after main FMR in the high-pressure film may have originated from the dominant PMA component, and disappeared when using a bias field. Furthermore, these multiple peaks were missing in the AC permeability spectra for low-pressure regime films (1.6–4 mTorr), see Fig. 6, which further confirms the dominant uniaxial anisotropy in low-pressure films, as seen in the BH loops (Fig. 4).

**Figure 8:**
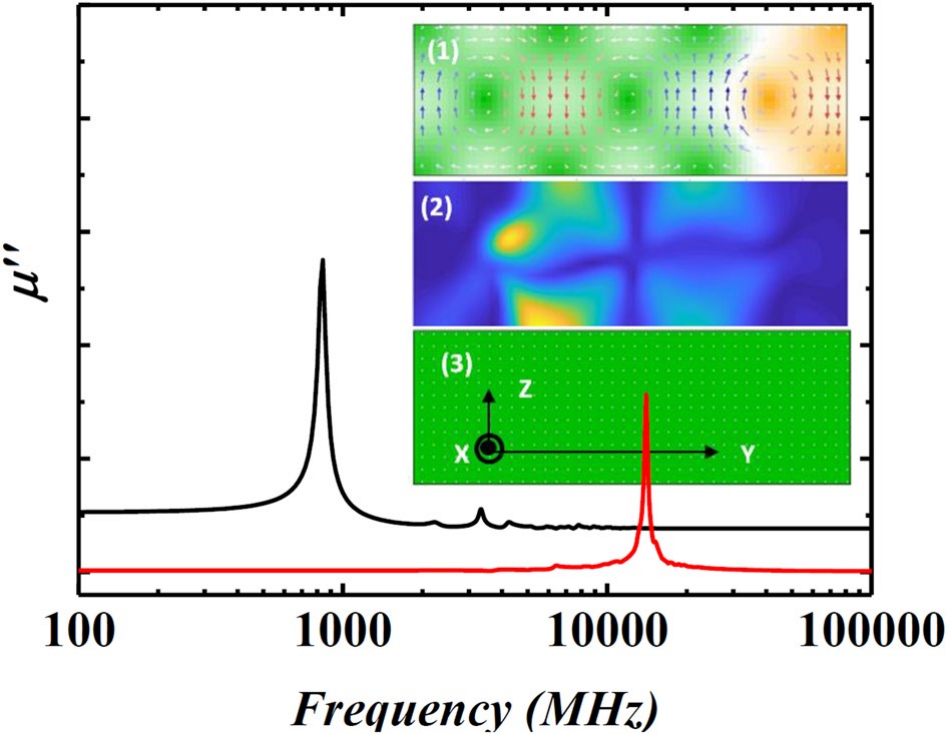
OOMMF simulation of the high-frequency imaginary permeability (*µʺ)* for films with different magnetisation configurations. The solid black line shows the simulated *µʺ* of the film with a dominant PMA, while the solid red line indicates the *µʺ* of the film with strong in-plane uniaxial anisotropy. In-plane easy axis is along the x-axis, the thickness is along the z-axis, and the configuration is periodic along the y-axis. Inset 1 shows the cross-section of the static magnetisation configuration (arrows indicate the magnetisation direction, the green colour indicates the magnetisation direction along the positive x-axis, the orange colour shows the magnetisation along the negative x-axis). Inset 2 shows the FMR magnitude distribution (red colour indicates a larger magnitude, the blue colour indicates a smaller magnitude). Inset 3 shows the static magnetisation configuration of the films. All inset images were generated using OOMMF micromagnetic software (version 1.2 (beta), http://math.nist.gov/oommf).

Furthermore, OOMMF micromagnetic simulations^44^ were performed to verify the effect of PMA on the emergence of multiple peaks after the main FMR. 2D static micromagnetic simulations (78 × 25 grids) were constructed for the film thickness 250 nm with, (1) a dominant PMA for the film deposited under 6.2 mTorr (Fig. 8, Inset 1), and (2) a significant in-plane uniaxial anisotropy for the film with strong biased field (Fig. 8, Inset 3), following the method introduced by Youssef et al.^45^. The in-plane easy axis is along the stripe domains and defined as the x-axis, the thickness is defined along the z-axis, and periodic structures with vortex-like domain walls were constructed along the y-axis. The PMA was set as 0.3 × 10^5^ erg/cm^3^ for films deposited under 6.2 mTorr, which results in a domain width ~ 200 nm comparable to the domain width as seen in the MFM image presented in Fig. 5c. In this zero-field cross-sectional equilibrium magnetisation configuration, m_x_ was considered as 0.57, where m_x_ is the ratio of magnetic remanence over magnetic saturation. This is comparable with the BH loop measurements for the high-pressure film. The *µʺ* of the zero-field microwave permeability (excited along the y-axis) were simulated, which showed multi-peaks after the main FMR (Fig. 8, solid black line), similar to the experimental results presented in Fig. 6b. The Fig. 8 inset 2 shows the magnitude distribution at the secondary peak position, where the red colour indicates a larger magnitude and the blue colour indicates a smaller magnitude, which shows that the secondary peaks are mainly contributed by localized resonances, similar to the work reported elsewhere^45^. While the simulation results for films with strong in-plane uniaxial anisotropy (6.2 mTorr films with strong in-plane biased field) only show a single FMR peak, shown in Fig. 8 (solid red line), similar to the result under > 70 Oe biased field in Fig. 7. Conclusively, simulation results further confirm that multiple peaks in high-pressure films emerged due to the dominant PMA component, which disappeared when PMA became feeble due to applying a suitable in-plane biased field.

The PMA shows the detrimental effects on the high-frequency soft magnetic properties of films, such as high *H*_*c*_, low permeability, and high material loss due to multimode resonance in the permeability spectrum, as discussed in detail in previous sections. Several mechanisms have been proposed as an origin of PMA in amorphous thin films. For example, Sharma et al.^14^ concluded that the residual stress and high degree of atomic randomness are the fundamental reasons for the emergence of the PMA in Co–Fe–B–Ta amorphous films. The residual stress of each film was measured by calculating the difference in the curvature of the wafer pre- and post-deposition of the films. The stress values remained in the range of 0.15–0.27 GPa for the series of the investigated films, though no systematic change was observed. All films revealed compressive stress in nature where the films stretch after deposition. The target alloy used for sputtering was in direct line of sight with the substrate rather than utilising oblique angle deposition, which tends to promote the formation of tensile stress due to the increase in inter-columnar voids^46^. A long mean free path due to low deposition pressure results in the sputtered atoms arriving at the substrate at near-normal incidence, and compressive type stress is typically seen under this mode^47^. One would expect the stress of the high-pressure film of 6.2 mTorr to be tensile in nature due to the increased incident angle of arriving atoms. However, the existence of porous inter-columnar voids, see Fig. 3b, can reduce the effect of tensile stress^32^. The incorporation of oxygen from the surrounding air after removing the film from the vacuum chamber nullifies the tensile stress, and eventually, induces compressive residual stress^48^. In addition, porous films are also susceptible to impurity formation when exposed to ambient atmospheric conditions after deposition. This might be the reason for the white dot contaminants, as seen in the AFM image of the 6.2 mTorr films in Fig. 2i. Conclusively, the residual stress of all films remained compressive in nature and comparable in magnitude, suggesting that it should not be the dominant factor to influence the underlying mechanism of magnetism.

A correlation between thin film growth mechanism from a homogeneous disorder atomic structure to a porous inter-columnar void, the emergence of PMA from a feeble strength to a dominant component, and evolution of single-mode FMR to a multimode resonance suggests the origin of perpendicular magnetisation in the high-pressure film is related to the remarkable shape anisotropy in out-of-plane direction originated by the columnar growth of the films. To the author’s best knowledge, this is the ever first report that links the high sputtering pressure to the columnar structure of the films as a mechanism of perpendicular magnetisation in amorphous thin films.

## Conclusions

The Co–Zr–Ta–B amorphous films of 250 nm thickness were deposited using DC magnetron sputtering from a single alloy target. The effect of argon sputtering pressure (1.6–6.2 mTorr) on the thin film growth mechanisms, global magnetic behaviours, magnetic domains, and high-frequency permeability response was investigated. The structural analysis of the films confirmed the amorphous nature of the atomic structure at the local scale. Thin-film growth mechanism drastically changed from a homogeneous structure to a porous columnar structure when the deposition pressure elevated from a low (1.6 mTorr) to a high-pressure (6.2 mTorr) regime. The columnar growth of the films was ascribed to the lower energy of sputtered atoms arriving at the substrate and a self-shadowing effect. The films deposited under low-pressure retained dominant in-plane uniaxial magnetic anisotropy, though an incremental increase in the PMA component as a function of deposition pressure was observed, as confirmed by the evolving BH loops and faint stripe domain patterns. The anisotropy of the films deposited under high-pressure completely transformed to perpendicular, thus resulting in an out-of-plane magnetic configuration, as confirmed by isotropic transcritical BH loops, stripe magnetic domain pattern, and multiple mode resonance in high-frequency permeability spectrum. The OOMMF micromagnetic simulations further confirmed the origin of multiple resonance peaks was associated with the dominant PMA in high-pressure films. We conclude that the origin of PMA in the present amorphous films is associated with porous columnar microstructure, grown due to the high sputter deposition pressure. Thus, we suggest that the ultra-soft magnetic performance of amorphous films can be retained by carefully selecting a low-pressure deposition regime to avoid the porous columnar growth of films.

## Methods

Amorphous films of Co_84_–Zr_4_–Ta_4_–B_8_ (atomic %) alloy were deposited by a DC-magnetron sputtering (Nordiko Advanced Energy NDX 2500-W) deposition technique. DC magnetron sputtering was used on an 8″ single alloy target (Testbourne Ltd., 99.9% purity) with a throw distance of 5.5 cm. The same alloy target was used for all deposition runs. The sputtering chamber was pumped down to a base pressure of ~ 10^–7^ mbar. Prior to deposition, the substrates (100 mm diameter Si/SiO_2_, 0.25 um thermal oxide) were cleaned by generating an RF plasma in the sputtering chamber at 1 kW for 25 min using high purity argon gas. An adhesive layer of 20 nm thickness of Ti was deposited prior to the deposition of the magnetic film. An aligning magnetic field was used during deposition, and a DC power of 1 kW was applied to the target material. The deposition was in a bottom-up configuration and the wafers were sitting on a carousel. The carousel rotation throughout the deposition was kept constant at 10 revolutions per minute (RPM). A Dektak profilometer was used to determine the stress and thickness of each film. The deposition rate was calibrated at 22.7 nm/min by measuring the step height of the films. The thickness of the magnetic material was kept constant at 250 nm for the whole series of films. The flow rate of the argon gas was adjusted using a mass flow controller before every deposition run to achieve the desired argon pressure for the sputtering of the magnetic alloy. Seven films were deposited under the same deposition parameters except varying the argon pressure in the range of 1.6–6.2 mTorr.

XRD was used to determine the atomic structure of each film (XRD, Phillips Xpert diffractometer, Cu Kα − 1.54 Å). Surface morphology images were taken with a SEM and AFM (Bruker Dimension Icon). The solidus temperature of the Co–Zr–Ta–B alloy was found using Pandat software (version 2019, https://computherm.com), based on the thermodynamics database. The temperature of the substrate during deposition was measured using temperature-sensitive labels (Radionics). TEM lamella cross-sections were prepared using a DualBeam FIB (FEI Helios NanoLab 600i). The microstructural analysis was performed using a high-resolution transmission electron microscope (HR-TEM, Jeol 2100, 200 kV) using a double tilt sample holder. The resistivity of each film was measured using a 4-point probe setup and the resistivity of the titanium layer was subtracted in each case. The static magnetic properties were investigated by using a B-H loop tracer (SHB, MESA 200 HF) on the 100 mm wafer. Magnetic domain images were taken over a 10 µm × 10 µm area using MFM (Bruker Dimension Icon) at the remnant state of the magnetisation along with surface topography images. A high-frequency Permeameter (Ryowa, PMM 9G) was used to measure the permeability of 4 mm × 4 mm samples taken from the centre of each wafer. Micromagnetic simulations were undertaken using OOMMF micromagnetic software (version 1.2 (beta), http://math.nist.gov/oommf).
